# Global Transcriptomic Profiling Using Small Volumes of Whole Blood: A Cost-Effective Method for Translational Genomic Biomarker Identification in Small Animals

**DOI:** 10.3390/ijms12042502

**Published:** 2011-04-13

**Authors:** Meagan M. Fricano, Amy C. Ditewig, Paul M. Jung, Michael J. Liguori, Eric A. G. Blomme, Yi Yang

**Affiliations:** Global Pharmaceutical Research and Development, Abbott Laboratories, 100 Abbott Park Road, Abbott Park, Il 60064, USA; E-Mails: meagan.fricano@abbott.com (M.M.F.); amy.ditewig@abbott.com (A.C.D.); paul.jung@abbott.com (P.M.J.); michael.liguori@abbott.com (M.J.L.); eric.blomme@abbott.com (E.A.G.B.)

**Keywords:** blood, biomarker, inflammation, transcriptomics

## Abstract

Blood is an ideal tissue for the identification of novel genomic biomarkers for toxicity or efficacy. However, using blood for transcriptomic profiling presents significant technical challenges due to the transcriptomic changes induced by *ex vivo* handling and the interference of highly abundant globin mRNA. Most whole blood RNA stabilization and isolation methods also require significant volumes of blood, limiting their effective use in small animal species, such as rodents. To overcome these challenges, a QIAzol-based RNA stabilization and isolation method (QSI) was developed to isolate sufficient amounts of high quality total RNA from 25 to 500 μL of rat whole blood. The method was compared to the standard PAXgene Blood RNA System using blood collected from rats exposed to saline or lipopolysaccharide (LPS). The QSI method yielded an average of 54 ng total RNA per μL of rat whole blood with an average RNA Integrity Number (RIN) of 9, a performance comparable with the standard PAXgene method. Total RNA samples were further processed using the NuGEN Ovation Whole Blood Solution system and cDNA was hybridized to Affymetrix Rat Genome 230 2.0 Arrays. The microarray QC parameters using RNA isolated with the QSI method were within the acceptable range for microarray analysis. The transcriptomic profiles were highly correlated with those using RNA isolated with the PAXgene method and were consistent with expected LPS-induced inflammatory responses. The present study demonstrated that the QSI method coupled with NuGEN Ovation Whole Blood Solution system is cost-effective and particularly suitable for transcriptomic profiling of minimal volumes of whole blood, typical of those obtained with small animal species.

## 1. Introduction

Global transcriptomic profiling is a useful tool to identify novel biomarkers for disease and toxicity [1–4]. To date, most genomic biomarkers have been identified using solid tissues, such as liver, kidney, or neoplastic masses. However, it is usually impractical to obtain tissue biopsies in clinical studies or for continuous pre-clinical monitoring.

Peripheral blood has obvious advantages for biomarker discovery due to its non-invasive collection and availability. In addition, biomarkers identified in blood should be easily translated from preclinical species to humans. A number of studies have demonstrated that transcriptomic changes in peripheral blood can serve as biomarkers of exposure to xenobiotics or as biomarkers for pathological changes occurring in other tissues [5–8].

Peripheral blood mononuclear cells (PMBCs) can be used for genomics biomarker studies since they are the most transcriptionally active cells in blood. However, the extra fractionation procedure required for PBMCs can have a significant impact on the transcriptome [9]. Isolation of PBMCs at the time of blood collection can also be a major limitation in large clinical trials as the procedure requires skilled technicians and specialized equipment. As a result, whole blood becomes a more attractive sample for blood-based genomic biomarker discovery. However, the use of whole blood for transcriptomic profiling presents a number of significant technical challenges.

First, RNA degradation and transcriptomic changes can occur quickly after the blood is drawn from subjects [10,11]. Traditional reagents, such as citrate salts, heparin, and EDTA, inhibit blood clotting, but do not stabilize mRNA transcripts [12]. Up-regulation of genes related to hypoxia and down-regulation of genes related to metabolism and cell cycle have been observed in whole blood samples when RNA was not immediately isolated after blood collection [11].

Attempts to overcome these hurdles led to the development of a number of new approaches for blood RNA stabilization [13]. One of those is the PAXgene blood RNA system that has been widely used in clinical settings for blood transcriptomic studies. The PAXgene system uses a proprietary reagent that stabilizes RNA immediately upon blood collection, making it possible to store samples for relatively long periods of time without compromising RNA integrity [12,14]. Although this system allows for easy blood collection, storage, and transport, its use in pre-clinical settings is significantly limited by its volume requirement. The PAXgene Blood RNA System is designed for the isolation of total RNA from 2.5 mL human whole blood. In animal models, particularly in rodents, such volumes are not usually achievable even at terminal blood draws especially when other parameters, such as clinical chemistry, need to be evaluated in parallel.

The second major concern for whole blood transcriptomic analysis is the interference from globin mRNA. Whole blood contains abundant α- and β-globin mRNA transcripts that comprise up to 70% of total mRNA [15]. Such a high abundance of globin mRNA can significantly mask the signal of low abundance transcripts as a result of non-specific cross-hybridization with non-globin transcripts [16,17]. Technologies to reduce globin mRNA, such as GLOBINclear (Ambion, Applied Biosystems) or globin peptide nucleic acid (PNA) oligos, are time-consuming, low throughput, and prone to additional experimental variability [17]. Although the globin reduction procedures can efficiently increase the overall sensitivity of transcript detection, they may also reduce the signal intensities for other transcripts [16,18,19].

In order to overcome these challenges, we have developed a simple whole blood processing method that immediately stabilizes RNA upon blood collection and yields high quality RNA from as little as 25 μL of rat whole blood. To circumvent the need for a separate globin reduction procedure, the NuGEN Ovation Whole Blood Solution was employed for microarray sample preparation. The NuGEN approach hybridizes biotinylated cDNA onto the microarray and has been shown to have sufficient sensitivity and reproducibility with small amounts of input RNA [17]. Using blood from rats exposed to lipopolysaccharide (LPS) as a model system, the present study demonstrates that the RNA isolated by the proposed QIAzol-based stabilization and isolation method (QSI) can provide robust transcriptomic profiles comparable to those obtained from RNA isolated with the standard PAXgene system.

## 2. Results and Discussion

### 2.1. Results

#### 2.1.1. Quantity and Quality Assessment of Total RNA Isolated from Rat Whole Blood Using the PAXgene and QSI Methods

Total RNA was isolated from 2.5 mL rat whole blood using the standard PAXgene method or from 500 μL rat whole blood using the QSI method. When normalized with blood volume, the total RNA yield from the PAXgene method was approximately twice the yield from the QSI method (Table 1).

Given the small amount of input RNA required for NuGEN Whole Blood Solution, both isolation methods yielded a sufficient amount of RNA for microarray analysis. RNA quality from both isolation methods was excellent with an average RIN score of 9.

#### 2.1.2. RNA Isolated from the PAXgene and QSI Methods Yield Comparable Affymetrix GeneChip® Array Performance

The array performance metrics are summarized in Table 2. The percent present calls (% *P*) for the RNA samples isolated using the QSI method were generally lower than those produced from the PAXgene method (*p*-value < 0.05). However, there was no significant difference in the GAPDH 3′/5′ ratios and β-actin 3′/5′ ratios between the PAXgene and the QSI methods.

#### 2.1.3. The PAXgene and QSI Methods Yield Comparable Blood Transcriptomic Profiles in Rats Exposed to LPS

**Figure 1.**
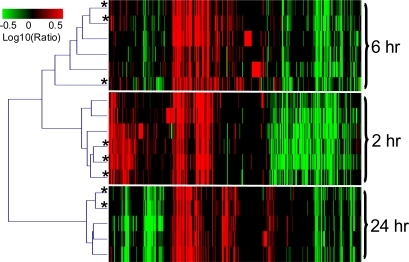
Agglomerative hierarchical clustering of gene expression profiles from blood samples of LPS-treated rats. Gene expression data were calculated by normalizing treatment to time-matched vehicles using the same RNA isolation method. Each column represents a single Affymetrix probe set and each row an experimental treatment. Only transcripts with a fold change higher than 2 fold and a *p*-value less than 0.01 in at least one ratio experiments are displayed (*n* = 6197). Increases in mRNA level are represented as shades of red and decreases as shades of green. If the p-value for a particular gene expression change was greater than 0.01, the log10(ratio) was represented as zero or black on the heat map. * denotes gene expression profiles from blood RNA samples isolated with the QSI method.

LPS induced the highest number of gene expression changes in rat blood at 2 hrs, followed by 6 hrs and 24 hrs. Figure 1 showed the agglomerative hierarchical clustering of gene expression profiles from blood samples of LPS-treated rats. The gene expression profiles were clustered according to treatment time, but not to the RNA isolation method. Furthermore, the variability introduced by the RNA isolation methods was no greater than the underlying biological variability among rats. For instance, the pair-wise correlation coefficients between the transcriptomic changes from any two rats treated with LPS for 2 hrs was 0.84 ± 0.02 using RNA isolated with the PAXgene method, and 0.87 ± 0.01 using RNA isolated with the QSI method. This was comparable to the pair-wise correlation coefficient between the transcriptomic changes using input RNA isolated by either methods from the same animal, which was 0.86 ± 0.01 (*p*-value = 0.44 and 0.43, respectively).

**Figure 2.**
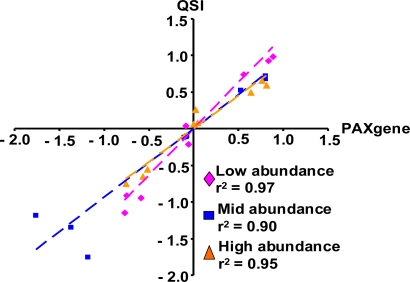
Effects of blood RNA isolation methods on gene transcripts of different abundance. The gene expression changes are compared between samples isolated with the PAXgene and QSI methods for selective gene transcripts with low, mid, and high basal gene expression levels. Data on both axes are expressed as the log10 gene expression ratios from the individual LPS-treated sample vs. the vehicle controls at the 2-hr time point.

To address whether blood RNA isolation methods could selectively affect transcripts of different abundance, the gene expression levels of a panel of nine transcripts representing various abundance levels were further evaluated using the 2-hr time point samples. The signal intensities of the low abundance transcripts were less than 3-fold of the microarray background signals. The intensity of the mid abundance transcripts were 14–26-fold above background levels, while the intensities of the high abundance transcripts were over 80-fold above background, representing the top 5th percentile of the rat genome. Each abundance category contained a transcript that was up-regulated, down-regulated, or unchanged (less than 2-fold relative to control treatments) by LPS treatment. The list of transcripts is provided in the supplemental table. As shown in Figure 2, the magnitudes of changes in gene expression from samples isolated with the QSI method were almost identical to the ones from the PAXgene isolation method. The r^2^ was over 0.9 in any abundance category.

**Figure 3.**
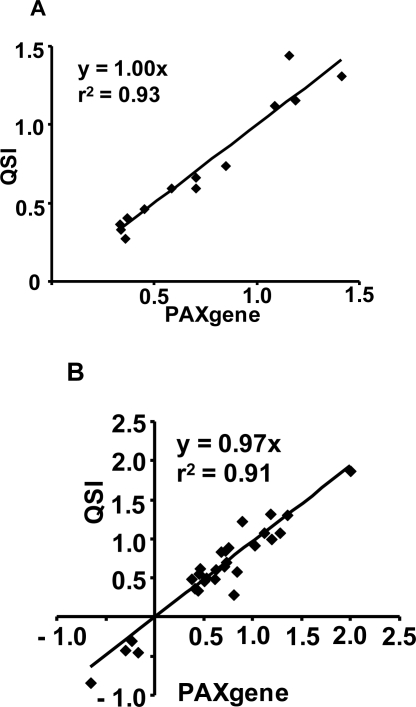
Effects of blood RNA isolation methods on genes in the interferon and IL-10 pathways. The gene expression changes are compared between samples isolated with the PAXgene and QSI methods for the genes in the interferon (**A**) and IL-10 (**B**) pathways. Data on both axes are expressed as the average log10 gene expression ratios from the LPS treated samples *vs.* the vehicle controls. Only genes with a fold change higher than 2 fold and *p*-value < 0.01 in either isolation methods are included in the analysis.

To evaluate the impact of the RNA isolation method on characterization of biological functions, blood transcriptomic profiles were also subjected to pathway analysis using Ingenuity Pathway Analysis Software. The analysis was focused on the samples from the 2-hr time point since they represented the peak transcriptomic changes induced by LPS. As expected, LPS induced gene expression changes consistent with perturbation of immune responses. Regardless of the RNA isolation method, the top five regulated physiological functions were: hematological system development and function, immune cell trafficking, cell-mediated immune response, and tissue morphology. In addition, the top five most impacted canonical signaling pathways were the same with the two RNA isolation methods (Table 3). Figure 3 shows a comparison of the gene expression data between the two isolation methods for the genes in the top two canonical pathways, namely interferon and IL-10 pathways. In both cases, the gene expression changes showed almost a 1:1 relationship with an *r*^2^ > 0.9.

#### 2.1.4. Quantitative RT-PCR Validation of Microarray Results

To confirm the microarray results, the expression levels of the same panel of 9 transcripts representing different abundance levels were measured by real-time RT-PCR using 2-hr blood RNA samples isolated with the PAXgene method. Consistent with the microarray results, the transcripts were shown to be regulated in the same direction for all abundance levels (Table 4). The transcripts unchanged per microarray measurement were also shown to not be regulated by RT-PCR, except for the low abundance transcripts which fluctuated further away from vehicle control when measured by RT-PCR. However, given the low basal expression level, these changes are not likely to be biologically significant. These results indicated that the NuGEN Whole Blood Solution, while reducing the interference associated with highly abundant globin mRNA transcripts, did not selectively amplify genes according to their abundance.

#### 2.1.5. Evaluation of the QSI Method Using Small Volume of Whole Blood as Input

To assess if small blood volumes are adequate for transcriptomic profiling using the QSI method, various volumes of whole blood (25–200 μL) were collected from rats treated with saline or LPS for 2 hrs. Transcriptomic profiles were generated using the QSI method for RNA isolation followed by the NuGEN method for RNA amplification and target labeling. When normalized with blood volume, the smaller amount of blood input yielded the greatest amount of total RNA produced by volume (Table 1). Furthermore, the QSI method using ≤ 100 μL blood yielded over 50% higher total RNA per μL of blood than the standard PAXgene method. The quality of RNA isolated from small volumes of blood, as represented by RIN score, was similar to that isolated from 500 μL whole blood (Table 1).

**Figure 4.**
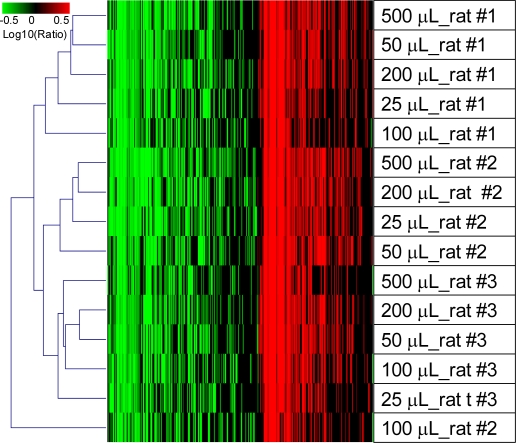
Effects of the starting blood volume on global transcriptomic profiles using the QSI method. Gene expression profiles from blood samples of rats exposed to LPS for 2 hrs were clustered using the agglomerative hierarchical algorithm. RNA was isolated using different volumes of whole blood with the QSI method. Gene expression data were calculated by normalizing treatment to the vehicles using RNA isolated from the same volume of whole blood. Each column represents a single Affymetrix probe set and each row an experimental treatment. Only genes with a fold change higher than 2 fold and *p*-value < 0.01 in at least one experiment are displayed (*n* = 4406). Increases in mRNA level are represented as shades of red and decreases as shades of green. If the *p*-value for a particular gene expression change was greater than 0.01, the log10 (ratio) was represented as zero or black on the heat map. Note the marked similarity in expression profiles indicating that the starting blood volume did not significantly influence microarray data.

There were no significant differences in microarray performance using RNA isolated from various volumes of blood (Table 2). Figure 4 shows the whole blood transcriptomic profiles from the LPS-treated rats using RNA isolated from different volumes of whole blood.

**Figure 5.**
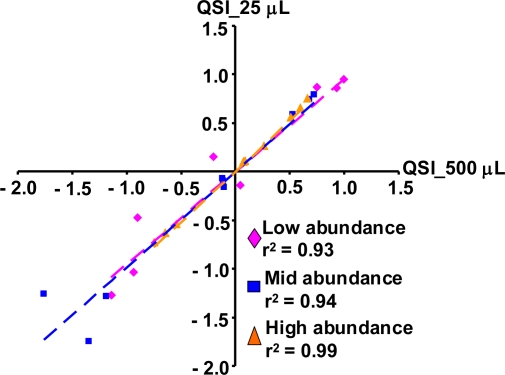
Effects of the starting blood volume on gene transcripts of different abundance. The gene expression changes were compared between samples prepared from 500 uL and 25 uL whole blood for selective gene transcripts with low, mid, and high basal gene expression levels. Data on both axes are expressed as the log10 gene expression ratios from the individual LPS treated sample vs. the vehicle controls at the 2-hr time point.

In general, the gene expression profiles were clustered by individual animal, not by blood volume. The impact of blood volume on specific transcript panels and biological functions were further evaluated using samples isolated from 500 μL or 25 μL whole blood. The gene expression levels from the panel of 9 transcripts representing different abundance levels were highly correlated with an *r*^2^ > 0.9 between the two sets of samples (Figure 5). The same top five canonical signaling pathways were impacted to a similar extent regardless of the starting blood volume (Table 3).

### 2.2. Discussion

Identification of blood genomic biomarkers of efficacy or toxicity in small preclinical species, especially rodents, offers great potential for translation to the clinic. However, the sample collection, RNA isolation procedures, and microarray processing methods need to be optimized to small amount of whole blood in order to make such research activity practical in animal models. The present study describes a practical and efficient workflow using a QSI isolation method coupled with NuGEN Ovation Whole Blood Solution for blood transcriptomic profiling from as little as 25 μL whole blood, a volume easily obtained from a standard tail vein bleeding procedure. A comparison with the standard PAXgene system demonstrated that the QSI method can produce similar yields of total RNA per volume of rat whole blood. The total RNA samples produced from the QSI and the standard PAXgene methods have an average *RIN* score of 9, well above the RNA quality generally preferred for microarray-based profiling (*RIN* > 8) [10,20,21].

It is possible to modify the standard PAXgene method to isolate RNA from small amounts of whole blood. The method by Krawiec *et al.* [21] yielded an average of 40 ng total RNA per μL whole blood with a *RIN* score of 7.7 from 50 μL mouse whole blood. The method by Robison *et al.* [22] yielded an average of 3.6 ng total RNA per μL whole blood with a RIN score of 9.3 from 70 μL whole human blood collected via fingerstick. The low yield from this later study is likely a reflection of the difference between human and rodent blood. In general, the RNA yield and quality were comparable to the QSI method reported here. However, both modified PAXgene methods are manual and require extra steps to achieve sufficient yield. In contrast, the QSI method can be fully automated. Using the Qiagen Automated BioRobot 3000 RNeasy-96 RNA isolation protocol, only 90 min of hands-on time is needed to isolate RNA from 96 whole blood samples. The QSI method is also cheaper than the PAXgene method. The total cost for 96 samples using the automated QSI method is a quarter of the cost of the standard PAXgene method and half the cost of the modified PAXgene method.

Commercial RNA isolation and stabilization kits that are designed specifically for laboratory animals have also been recently developed from several resources. The ZR Whole Blood Total RNA Kit (Zymo Research, Orange, CA, USA), the Mouse RiboPure Blood RNA Isolation Kit (Ambion/Applied Biosystems, Austin, TX, USA), and the RNeasy Protect Animal Blood System (Qiagen, Valencia, CA, USA) are designed for blood volumes of 100–500 μL. Whether these kits can be further scaled down to 25 μL whole blood remains to be determined.

Of note, neither the PAXgene system, nor the QSI method removes globin mRNA during the RNA isolation process. To reduce the artifacts associated with highly abundant globin mRNA transcripts, the NuGEN Ovation Whole Blood Solution procedure was used downstream of the total RNA isolation from rat whole blood. Results with human blood samples suggested that globin RNA amplification can be reduced with the NuGEN procedure [17]. However, in our experience with rat whole blood, there was no significant reduction of globin peak in cDNA samples when compared to cRNA samples prepared with the standard Affymetrix protocol. Similar results were also noted by the manufacturer (personal communication). Rat blood has twice the amount of reticulocytes compared to human blood [23]. The difference in globin reduction performance between human *vs.* rat blood is likely a reflection of the high proportion of reticulocytes in rat blood. The NuGEN procedure generates cDNA targets as compared to the cRNA targets prepared with the standard Affymetrix protocol. Despite the pronounced globin peak in cDNA targets from rat blood, the procedure is less prone to non-specific cross-hybridization with globin transcripts as a result of higher fidelity of DNA-DNA hybridization compared to RNA-DNA hybridization [24]. In addition to the reduced interference with globin mRNA, the NuGEN procedure requires a relatively small amount (20–50 ng) of total RNA for transcriptomic profiling. The QSI method described here can easily yield over 1000 ng total RNA from 25 uL blood, an amount sufficient for several microarray experiments using the NuGEN procedure.

A comparison of RT-PCR and microarray results showed good concordance of gene expression changes for a panel of 9 transcripts selected to represent low, mid, and high abundance genes. There are differences in the absolute fold change measured by RT-PCR and microarray. However, the trend (ie., up- or down-regulation) was the same across all abundance levels. The concordance in trend, but not in absolute value, is a typical observation between RT-PCR and any type of microarray platform regardless of sample resources [25,26]. Although this is only a small sample size relative to the whole genome, these results, taken together with the prototypical LPS-induced pathway changes in the blood transcriptome, suggested that the NuGEN procedure can provide an accurate representation of transcripts across different abundance levels.

The microarray performance metrics showed that the array quality using RNA prepared from the PAXgene method was slightly superior to that from RNA isolated with the QSI method. However, the QC parameters for all samples were within the acceptable range recommended by the manufacturer and the general microarray community [27–30].

We used LPS-induced acute immune response in rats as a model system to address if the slight difference in QC parameters between the two methods affected the representation of gene transcripts and biological interpretation. LPS is a component of the bacterial cell wall of Gram negative bacteria and activates a complex of pattern recognition proteins in a variety of mammalian cell types, especially macrophages [31]. The interaction with the protein complex results in activation of a downstream signaling cascade that eventually leads to the production of pro-inflammatory cytokines, as well as the recruitment of inflammatory cells. In human whole blood, LPS induces transcriptomic changes characterized by increased expression of genes associated with the defense response to pathogens, such as cytokines, chemokines, and acute-phase transcription factors, and decreased expression of genes associated with lymphocytes and ribosomal proteins [32]. In the present study, the rat blood transcriptomic profiles revealed a pattern consistent with the underlying LPS-induced acute immune response, regardless of whether the blood total RNA was isolated with the PAXgene or the QSI method.

## 3. Experimental Section

### 3.1. Animals, Treatment, and Sample Collection

Male Sprague-Dawley rats [Crl:CD^®^(SD)IGS BR] weighing approximately 250 g were obtained from Charles River Laboratories, Inc, Portage, MI, USA. The animals were permitted non-certified Rodent Chow and water *ad libitum*. Rats were housed two or three per cage for two days after receipt to aid in acclimation.

**Figure 6.**
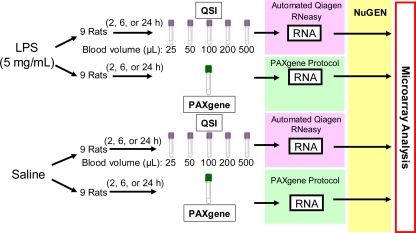
Flow diagram of the study design. Varying volumes of rat whole blood (25–500 μL) were collected and immediately lysed in QIAzol (25–200 μL of blood in 1 mL QIAzol, 500 μL in 2 mL QIAzol) from rats (*n* = 3 per time point per treatment) treated with either saline or LPS. Blood (2.5 mL) was also collected from each rat into PAXgene tubes. Automated RNA isolation for blood collected directly into QIAzol was performed using the Qiagen 3000 BioRobot RNeasy 96 protocol. RNA isolation for PAXgene tubes was performed manually according to manufacturer’s instructions.

Thereafter, rats were single housed in ventilated, stainless steel, wire bottom hanging cages equipped with feeders and an automatic watering system. The animals were administered a single intravenous injection of vehicle (saline) or LPS (5 mg/kg, Sigma-Aldrich # L2880) and euthanized 2, 6, or 24 hrs post dosing (3 rats/time point/group). Blood samples were collected via the abdominal vaudal vena cava at necropsy for RNA isolation (Figure 6). For the PAXgene method, 2.5 mL of blood were collected into PAXgene Blood RNA Tubes according to the manufacturer’s instructions (BD Biosciences, San Jose, CA). After incubation for 2 hrs at room temperature, the PAXgene tubes were stored at −20 °C until analysis. For the QSI method, approximately 25 μL, 50 μL, 100 μL, 200 μL, and 500 μL of blood were added to 1 mL or 2 mL (for 500 μL blood volume only) of QIAzol (QIAGEN, Valencia, CA). Upon addition of the blood, the tubes were inverted twice and incubated at room temperature for up to 3 hrs before storage at −80°C for future analysis. Experiments were conducted in accordance with the *Guiding Principles in the Use of Animals in Toxicology* [33] and approved by the local Institutional Animal Care and Use Committee.

### 3.2. RNA Isolation

RNA isolation from samples collected in PAXgene Blood RNA Tubes was performed according to the manufacturer’s specifications (BD Biosciences). For the QSI method, total RNA was isolated from 750 μL of blood-QIAzol lysate following the Qiagen Automated BioRobot 3000 RNeasy-96 RNA isolation protocol (QIAGEN). After manual centrifugation of a 96-well plate containing QIAzol lysate/chloroform mixture, the Qiagen BioRobot 3000 automatically transfers the upper, aqueous phase to a 96-well RNeasy plate for automated RNA purification. Nucleic acid concentration was determined by O.D. 260 nm (NanoDrop ND-1000, Thermo Scientific, Wilmington, DE, USA). The RNA integrity was evaluated and an RNA Integrity Number (RIN) was generated using an Agilent 2100 Bioanalyzer and its accompanying software (Agilent Technologies, Foster City, CA, USA).

### 3.3. Microarray Analysis

All blood RNA samples were processed to generate microarray hybridization targets using the Ovation Whole Blood Solution (NuGEN Technologies, San Carlos, CA, USA), a module containing three NuGEN products. Briefly, 50 ng of DNAase-treated RNA aliquots were amplified using the Ovation RNA Amplification System V2 coupled with the Ovation WB Reagent following the manufacturer’s instructions (NuGEN). Approximately 4.4 μg of each amplified single strand cDNA sample was then biotinylated and fragmented using the FL-Ovation cDNA Biotin Module V2. The resulting cDNA was hybridized to an Affymetrix Rat Genome 230 2.0 Array (Affymetrix, Santa Clara, CA, USA) using the NuGEN-modified hybridization protocol, with 2 min of heat denaturation of the hybridization cocktail at 99 °C and a minimum of 16 hrs of hybridization at 45 °C using an Affymetrix Hybridization Oven 640. The array was subsequently washed and stained with streptavidin-phycoerythrin (Molecular Probes) on the Affymetrix Gene-Chip Fluidics Workstation 400 following the NuGEN-recommended protocol EukGE-WS2v4_450. The array was then scanned using an Affymetrix GeneChip Scanner 3000.

### 3.4. Real-Time Quantitative RT-PCR

The following primers and probes were selected from the Applied Biosystems Assays-on-Demand gene expression products: *Tcf7* (Rn_00493446_m1), *Map2k6* (Rn_00586764_m1), *Nrp1* (Rn_00595457_m1), *Alas2* (Rn_00566201_m1), *Flad1* (Rn_01512487_m1), *Canx* (Rn_00596877_m1), S*100a9* (Rn0585879_m1), *Tnf* (Rn_99999017_m1), *Il1b* (Rn_01514151_m1), and *18S* (Hs_99999901_s1). Expression levels of each gene were quantified by real-time RT-PCR with an ABI Prism 7900 Sequence Detection System (Applied Biosystems, Carlsbad, CA, USA). Briefly, 200 ng of total RNA was reverse transcribed using the iScript^TM^ cDNA Synthesis Kit according to the manufacturer’s instructions (Bio-Rad Laboratories). Real-time PCR analyses were then performed in a total volume of 10 μL containing 1:15-diluted synthesized cDNA using Taqman^®^ Gene Expression Assays (Applied Biosystems). The PCR cycling profile was as follows: 10 min at 95 °C, followed by 40 cycles of 95 °C for 15 s and 60 °C for 1 min. The relative fold changes were calculated using the delta delta Ct method and normalized to 18S ribosomal RNA, the endogenous control gene.

### 3.5. Statistical Analysis

All non-microarray statistical analyses were conducted using JMP 7.0.1 statistical software from SAS (Cary, North Carolina). Comparisons were made using the two-tailed t-test with a significance value of *α* = 0.05. For microarray analysis, the scanned image and intensity files were imported into Rosetta Resolver gene expression analysis software version 7.2 (Rosetta Inpharmatics, Seattle, WA). Individual gene expression ratios were built for each treatment animal versus the averaged vehicle controls at the corresponding conditions using the Rosetta Resolver error model [34]. Genes and experimental treatments were grouped for visualization by agglomerative hierarchical clustering using the Pearson correlation as described in each figure. For pathway analysis, the average gene expression ratios were calculated as the *in silico* pool of treated animals versus the corresponding vehicle controls using the Rosetta Resolver error model. Genes with a fold change over 2 and a *p*-value < 0.01 were mapped to pathways and biological functions using Ingenuity Pathway Analysis (Ingenuity Systems, www.ingenuity.com).

## 4. Conclusions

In the present study, we have demonstrated that the QSI method can produce sufficient quantities of high quality RNA from small volumes (25–500 μL) of rat whole blood. Furthermore, the QSI method can generate microarray results comparable to the standard PAXgene method. Given the simplicity of this blood collection procedure and the minimal volume of blood required, the method can be easily applied for blood genomics profiling in pre-clinical species.

## Supplementary Material



## Figures and Tables

**Table 1.** Quantity and quality of total RNA isolated from rat whole blood using the PAXgene or QSI method.

**Method**	**RNA Yield (ng/μL whole blood)**	**RIN Score**
PAXgene	44.9 ± 5.6	9.0 ± 0.2
QSI_500	21.0 ± 1.3	8.8 ± 0.4
QSI_200	33.6 ± 5.4	9.5 ± 0.2

QSI_100	79.4 ± 7.0	8.5 ± 0.5
QSI_50	69.0 ± 6.3	8.8 ± 0.2
QSI_25	65.4 ± 6.0	8.8 ± 0.2

Samples were extracted from rats treated with saline or LPS for 2 hrs. Values are expressed as mean ± SEM (*n* = 6).

**Table 2.** GeneChip array performance matrices.

**Method**	**% Present**	**GAPDH 3′/5′ Ratio**	**Actin 3′/5′/Ratio**
PAXgene	47.5 ± 2.1	2.7 ± 0.03	8.9 ± 0.3
QSI_500	37.9 ± 3.6*	2.5 ± 0.11	8.9 ± 1.2
QSI_200	37.2 ± 2.3*	2.7 ± 0.08	8.4 ± 0.3
QSI_100	33.7 ± 5.1*	2.9 ± 0.15	10.1 ± 1.1
QSI_50	37.0 ± 3.1*	2.8 ± 0.12	9.8 ± 1.1
QSI_25	36.5 ± 4.4*	2.7 ± 0.14	8.5 ± 0.6

Samples were extracted from rats treated with saline or LPS for 2 hrs. Values are expressed as mean ± SEM (*n* = 6).

* Significant difference from the PAXgene method (*p* < 0.05).

**Table 3.** Top five canonical signaling pathways regulated in rat blood by LPS treatment for 2 hrs.

**Pathway**	**RNA Isolation Methods**	**Total # of Genes in Pathway**	# of Regulated Genes in Pathway*	**% Regulated**
Interferon Signaling	PAXgene	30	13	43
	QSI_500		12	40
	QSI_25		13	43
IL-10 Signaling	PAXgene	70	23	33
	QSI_500		26	37
	QSI_25		23	33
Activation of IRF by Cytosolic Pattern Recognition Receptors	PAXgene	74	23	31
	QSI_500		22	30
	QSI_25		25	34
Death Receptor Signaling	PAXgene	64	19	30
	QSI_500		18	28
	QSI_25		20	31
Role of PKR in interferon Induction and Antiviral Response	PAXgene	46	13	28
	QSI_500		14	30
	QSI_25		16	35

Genes were considered regulated if the average fold changes were greater than 2 fold over vehicle controls with *p*-value < 0.01.

**Table 4.** Quantitative RT-PCT validation of microarray results on selected gene transcripts.

**Gene Symbol**	**Abundance Level**	**Regulated by LPS according to Microarray**	**Fold Change over Vehicle**	
			**Microarray**	**RT-PCR**
*Tcf7*	High	Down	−4.25 ± 0.75	−7.74 ± 1.59
*Nrp1*	Mid	Down	−19.22 ± 4.13	−4.65 ± 0.27
*Map2k6*	Low	Down	−5.16 ± 0.64	−16.92 ± 4.63
*Alas2*	High	No Change	0.37 ± 0.69	0.55 ± 0.63
*Canx*	Mid	No Change	−1.22 ± 0.02	−1.10 ± 0.04
*Flad1*	Low	No Change	−1.23 ± 0.04	−2.77 ± 0.31
*S100a9*	High	Up	5.52 ± 0.62	16.70 ± 3.80
*Il1b*	Mid	Up	5.35 ± 0.99	9.19 ± 1.61
*Tnf*	Low	Up	6.04 ± 1.23	3.94 ± 0.76

Values are expressed as mean ± SEM from three animals treated with 5 mg/kg of LPS for 2 hrs. Blood RNA was isolated with the PAXgene method.

## Abbreviations:

QSI: QIAzol-based RNA stabilization and isolation method
LPS: lipopolysaccharide
RIN: RNA Integrity Number
PBMC: peripheral blood mononuclear cells
