# Supplementary material for: Global Transcriptomic Profiling Using Small Volumes of Whole Blood: A Cost-Effective Method for Translational Genomic Biomarker Identification in Small Animals
Source: Int J Mol Sci. 2011 Apr 13;12(4):2502–17. doi: 10.3390/ijms12042502 (PMC3127131; doi:10.3390/ijms12042502)
Supplement: Supplementary file 1 [file ijms-12-02502-s001.pdf]

Table S1. List of gene transcripts representing various abundance levels in rat blood.

Gene Symbol	Gene Title	Entrez Gene ID	Representative Public ID	Affymetrix Probe Set ID	ABI probe ID	Abundance
Tcf7	Transcription factor 7, T-cell specific	363595	AW251860	1376197_at	Rn_00493446_m1	High
Nrp1	Neuropilin 1	246331	BE116566	1373577_at	Rn_00595457_m1	Mid
Map2k6	mitogen-activated protein kinase kinase 6	114495	NM_053703	1387809_at	Rn_00586764_m1	Low
Alas2	aminolevulinate, delta-, synthase 2	25748	NM_013197	1367985_at	Rn_00566201_m1	High
Canx	calnexin	29144	AA848326	1388442_at	Rn_00596877_m1	Mid
Flad1	flavin adenine dinucleotide synthetase	751787	AI171855	1373758_at	Rn_01512487_m1	Low
S100a9	S100 calcium binding protein A9	94195	NM_053587	1387125_at	Rn0585879_m1	High
Il1b	interleukin 1 beta	24494	NM_031512	1398256_at	Rn_01514151_m1	Mid
Tnf	tumor necrosis factor (TNF superfamily, member 2)	24835	AA819227	1391384_at	Rn_99999017_m1	Low
